# 5-(2-Nitro-1-phenyl­but­yl)-4-phenyl-1,2,3-selenadiazole

**DOI:** 10.1107/S1600536812007027

**Published:** 2012-02-29

**Authors:** S. Sankari, P. Sugumar, P. Manisankar, S. Muthusubramanian, M. N. Ponnuswamy

**Affiliations:** a Department of Chemistry, Sri Sarada College for Women (Autonomus), Fairlands, Salem 600 016, India; b Centre of Advanced Study in Crystallography and Biophysics, University of Madras, Guindy Campus, Chennai 600 025, India; c Department of Industrial Chemistry, Alagappa University, Karaikudi 630 003, India; d School of Chemistry, Madurai Kamaraj University, Madurai 625 021, India

## Abstract

In the title compound, C_18_H_17_N_3_O_2_Se, the selenadiazole ring is planar [maximum deviation = 0.012 (2) Å for the ring C atom bearing the phenyl substituent]. The dihedral angle between the selenadiazole ring and the attached benzene ring is 46.5 (1)°. There is one short intra­molecular C—H⋯Se contact.

## Related literature

For general background to selenadiazole derivatives, see: El-Bahaie *et al.* (1990 ▶); El-Kashef *et al.* (1986 ▶); Kuroda *et al.* (2001 ▶); Khanna (2005 ▶); Padmavathi *et al.* (2002 ▶); Plano *et al.* (2010 ▶); Stadtman (1991 ▶).
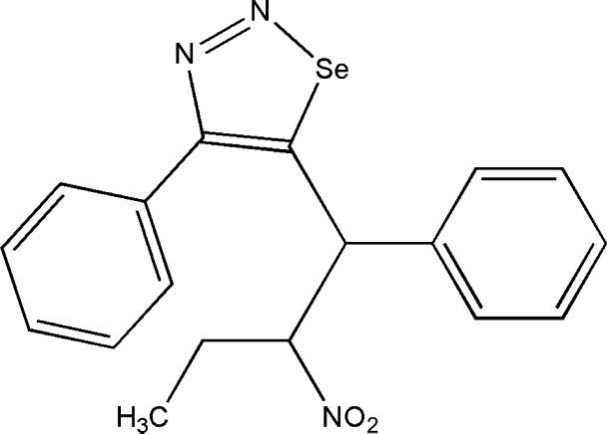


## Experimental

### 

#### Crystal data

C_18_H_17_N_3_O_2_Se
*M* *_r_* = 386.31
Triclinic, 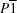

*a* = 7.879 (5) Å
*b* = 8.450 (5) Å
*c* = 13.438 (5) Å
α = 80.629 (5)°
β = 85.273 (5)°
γ = 75.352 (5)°
*V* = 853.2 (8) Å^3^
*Z* = 2
Mo *K*α radiation
μ = 2.22 mm^−1^
*T* = 293 K
0.20 × 0.18 × 0.16 mm

#### Data collection

Bruker SMART APEX CCD detector diffractometer
Absorption correction: multi-scan (*SADABS*; Bruker, 2008 ▶) *T*_min_ = 0.636, *T*_max_ = 0.702
15132 measured reflections
4265 independent reflections
3478 reflections with *I* > 2σ(*I*)
*R*_int_ = 0.027

#### Refinement

*R*[*F* ^2^ > 2σ(*F* ^2^)] = 0.032
*wR*(*F* ^2^) = 0.079
*S* = 1.05
4265 reflections
218 parameters
H-atom parameters constrained
Δρ_max_ = 0.43 e Å^−3^
Δρ_min_ = −0.21 e Å^−3^

### 

Data collection: *APEX2* (Bruker, 2008 ▶); cell refinement: *SAINT* (Bruker, 2008 ▶); data reduction: *SAINT*; program(s) used to solve structure: *SHELXS97* (Sheldrick, 2008 ▶); program(s) used to refine structure: *SHELXL97* (Sheldrick, 2008 ▶); molecular graphics: *ORTEP-3* (Farrugia, 1997 ▶); software used to prepare material for publication: *SHELXL97* and *PLATON* (Spek, 2009 ▶).

## Supplementary Material

Crystal structure: contains datablock(s) global, I. DOI: 10.1107/S1600536812007027/bt5784sup1.cif

Structure factors: contains datablock(s) I. DOI: 10.1107/S1600536812007027/bt5784Isup2.hkl

Supplementary material file. DOI: 10.1107/S1600536812007027/bt5784Isup3.cml

Additional supplementary materials:  crystallographic information; 3D view; checkCIF report

## Figures and Tables

**Table 1:** Hydrogen-bond geometry (Å, °)

*D*—H⋯*A*	*D*—H	H⋯*A*	*D*⋯*A*	*D*—H⋯*A*
C16—H16⋯Se1	0.98	2.85	3.313 (3)	110

**Fig. 1.**
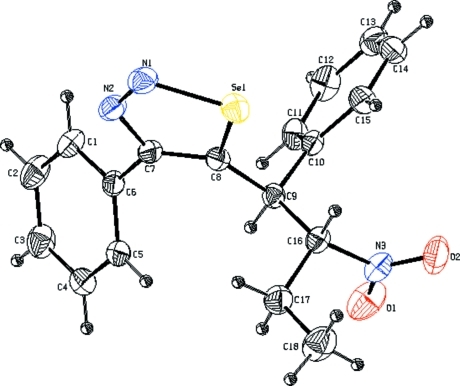
The molecular structure of the title compound, showing the atomic numbering and displacement ellipsoids drawn at 30% probability level.

**Fig. 2.**
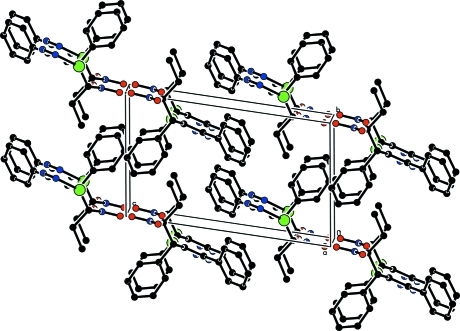
The crystal packing of the molecules viewed down a axis.

## supplementary crystallographic information

### Comment

Selenadiazoles, having one selenium and two nitrogen atoms in a five membered ring, are the important class of organoselenium compounds utilized in the synthesis of semiconductor nanoparticles (Khanna, 2005). These 1,2, 3-selenadiazoles are used as the synthetic intermediates in the preparation of many alkynes and other selenium compounds. In addition, 1,2,3-Selenadiazoles are of interest owing to their chemical properties and biological applications such as anti-fungal (Kuroda *et al.*, 2001), anti-bacterial (El-Kashef *et al.*, 1986), anti-microbial (El-Bahaie *et al.*, 1990), anti-cancer (Plano *et al.*, 2010) and insecticidal (Padmavathi *et al.*, 2002) properties. Glutathione peroxidases(GPx) are the antioxidant selenoenzymes protecting various organisms from oxidative stress by catalyzing the reduction of hydroperoxides at the expense of glutathione(GSH) (Stadtman, 1991). Owing to the above mentioned important properties of selenium containing compounds, the crystal structure of the title compound has been carried out.

The *ORTEP* plot of the molecule is shown in Fig.1. The bond lengths [Se1—N1] 1.877 (2)Å and [Se1—C8] 1.839 (2)Å are normal. The selenadiazol ring is planar and oriented at an angle of 46.5 (1)° with the attached phenyl ring. The phenylbutyl group is in extended conformation, which can be seen from the torsion angle values of [C9—C16—C17—C18]-178.5 (2)° & [C10—C9—C16—C17]-168.8 (2)°. The planar nitro group is oriented at an angle of 78.9 (2)° with phenylbutyl group. The molecular packing is controlled by C—H···π type of intermolecular interactions in addition to van der Waals forces.

### Experimental

A mixture of 4-nitro-1,3-diiphenylhexan-1-one (1 mmol), semicarbazide hydrochloride(2 mmol) and anhydrous sodium acetate (3 mmol) in ethanol (10 ml) was refluxed for 4 h. After completion of the reaction as monitored by TLC, the mixture was poured into ice cold water and the resulting semicarbazone was filtered off. Then, a mixture of semicarbazone (1 mmol) and SeO2 (2 mmol) in tetrahydrofuran (10 ml) were refluxed on a water bath for 1 h. The selenium deposited on cooling was removed by filtration, and the filtrate was poured into crushed ice, extracted with dichloromethane, and purified by column chromatography using silica gel (60–120 mesh) with 97:3 petroleum ether: ethyl acetate as eluent to give 5-(2-nitro-1-phenylbutyl)-4-phenyl-1,2, 3-selenadiazole.

### Refinement

H atoms were positioned geometrically with C—H = 0.93–0.98 Å and allowed to ride on their parent atoms,with *U*_iso_(H) = 1.5*U*_eq_(C) for methyl H and 1.2*U*_eq_(C) for other H atoms.

### Crystal data

**Table:**

C_18_H_17_N_3_O_2_Se	*Z* = 2
*M**_r_* = 386.31	*F*(000) = 392
Triclinic, *P*1	*D*_x_ = 1.504 Mg m^−^^3^
Hall symbol: -P 1	Mo *K*α radiation, λ = 0.71073 Å
*a* = 7.879 (5) Å	Cell parameters from 3478 reflections
*b* = 8.450 (5) Å	θ = 1.5–28.4°
*c* = 13.438 (5) Å	µ = 2.22 mm^−^^1^
α = 80.629 (5)°	*T* = 293 K
β = 85.273 (5)°	Block, white crystalline
γ = 75.352 (5)°	0.20 × 0.18 × 0.16 mm
*V* = 853.2 (8) Å^3^	

### Data collection

**Table:**

Bruker SMART APEX CCD detector diffractometer	4265 independent reflections
Radiation source: fine-focus sealed tube	3478 reflections with *I* > 2σ(*I*)
Graphite monochromator	*R*_int_ = 0.027
ω scans	θ_max_ = 28.4°, θ_min_ = 1.5°
Absorption correction: multi-scan (*SADABS*; Bruker, 2008)	*h* = −10→10
*T*_min_ = 0.636, *T*_max_ = 0.702	*k* = −11→11
15132 measured reflections	*l* = −17→17

### Refinement

**Table:**

Refinement on *F*^2^	Primary atom site location: structure-invariant direct methods
Least-squares matrix: full	Secondary atom site location: difference Fourier map
*R*[*F*^2^ > 2σ(*F*^2^)] = 0.032	Hydrogen site location: inferred from neighbouring sites
*wR*(*F*^2^) = 0.079	H-atom parameters constrained
*S* = 1.05	*w* = 1/[σ^2^(*F*_o_^2^) + (0.0382*P*)^2^ + 0.1656*P*] where *P* = (*F*_o_^2^ + 2*F*_c_^2^)/3
4265 reflections	(Δ/σ)_max_ = 0.001
218 parameters	Δρ_max_ = 0.43 e Å^−^^3^
0 restraints	Δρ_min_ = −0.21 e Å^−^^3^

### Special details

**Table:**

Geometry. All e.s.d.'s (except the e.s.d. in the dihedral angle between two l.s. planes) are estimated using the full covariance matrix. The cell e.s.d.'s are taken into account individually in the estimation of e.s.d.'s in distances, angles and torsion angles; correlations between e.s.d.'s in cell parameters are only used when they are defined by crystal symmetry. An approximate (isotropic) treatment of cell e.s.d.'s is used for estimating e.s.d.'s involving l.s. planes.
Refinement. Refinement of *F*^2^ against ALL reflections. The weighted *R*-factor *wR* and goodness of fit *S* are based on *F*^2^, conventional *R*-factors *R* are based on *F*, with *F* set to zero for negative *F*^2^. The threshold expression of *F*^2^ > σ(*F*^2^) is used only for calculating *R*-factors(gt) *etc*. and is not relevant to the choice of reflections for refinement. *R*-factors based on *F*^2^ are statistically about twice as large as those based on *F*, and *R*- factors based on ALL data will be even larger.

### Fractional atomic coordinates and isotropic or equivalent isotropic displacement parameters (Å^2^)

**Table:**

	*x*	*y*	*z*	*U*_iso_*/*U*_eq_	
C1	0.7979 (3)	0.3866 (2)	0.50318 (16)	0.0513 (5)	
H1	0.8590	0.4685	0.4849	0.062*	
C2	0.6659 (3)	0.4053 (3)	0.57649 (18)	0.0634 (6)	
H2	0.6391	0.4991	0.6082	0.076*	
C3	0.5726 (3)	0.2865 (3)	0.60360 (17)	0.0621 (6)	
H3	0.4822	0.3005	0.6529	0.074*	
C4	0.6131 (3)	0.1475 (3)	0.55777 (15)	0.0535 (5)	
H4	0.5500	0.0671	0.5760	0.064*	
C5	0.7476 (3)	0.1260 (2)	0.48458 (14)	0.0472 (4)	
H5	0.7754	0.0305	0.4545	0.057*	
C6	0.8415 (2)	0.2461 (2)	0.45570 (13)	0.0405 (4)	
C7	0.9868 (2)	0.2266 (2)	0.37864 (13)	0.0402 (4)	
C8	0.9887 (2)	0.1804 (2)	0.28553 (13)	0.0386 (4)	
C9	0.8345 (2)	0.1554 (2)	0.23419 (12)	0.0375 (4)	
H9	0.7444	0.1390	0.2869	0.045*	
C10	0.7575 (2)	0.3138 (2)	0.16406 (13)	0.0374 (4)	
C11	0.6121 (2)	0.4253 (3)	0.19758 (15)	0.0504 (5)	
H11	0.5577	0.3986	0.2600	0.060*	
C12	0.5464 (3)	0.5762 (3)	0.13925 (18)	0.0604 (6)	
H12	0.4495	0.6507	0.1632	0.073*	
C13	0.6231 (3)	0.6164 (3)	0.04674 (18)	0.0574 (5)	
H13	0.5790	0.7181	0.0078	0.069*	
C14	0.7656 (3)	0.5060 (3)	0.01180 (16)	0.0542 (5)	
H14	0.8170	0.5324	−0.0515	0.065*	
C15	0.8332 (2)	0.3560 (2)	0.06965 (14)	0.0467 (4)	
H15	0.9304	0.2824	0.0452	0.056*	
C16	0.8902 (2)	−0.0021 (2)	0.18459 (14)	0.0413 (4)	
H16	0.9943	0.0027	0.1401	0.050*	
C17	0.9315 (3)	−0.1575 (2)	0.26163 (16)	0.0581 (5)	
H17A	0.8273	−0.1624	0.3047	0.070*	
H17B	1.0224	−0.1500	0.3039	0.070*	
C18	0.9917 (4)	−0.3167 (3)	0.2162 (2)	0.0771 (7)	
H18A	0.8991	−0.3299	0.1786	0.116*	
H18B	1.0210	−0.4087	0.2693	0.116*	
H18C	1.0932	−0.3123	0.1721	0.116*	
N1	1.2673 (2)	0.2439 (2)	0.33702 (14)	0.0551 (4)	
N2	1.1401 (2)	0.26173 (19)	0.40163 (13)	0.0496 (4)	
N3	0.7421 (2)	−0.0094 (2)	0.12234 (14)	0.0520 (4)	
O1	0.6009 (2)	−0.0054 (2)	0.16418 (16)	0.0848 (6)	
O2	0.7744 (3)	−0.0209 (2)	0.03327 (13)	0.0806 (5)	
Se1	1.20608 (2)	0.17348 (3)	0.222464 (15)	0.05307 (9)	

### Atomic displacement parameters (Å^2^)

**Table:**

	*U*^11^	*U*^22^	*U*^33^	*U*^12^	*U*^13^	*U*^23^
C1	0.0508 (11)	0.0522 (11)	0.0580 (12)	−0.0167 (9)	−0.0044 (9)	−0.0211 (9)
C2	0.0607 (13)	0.0703 (14)	0.0679 (14)	−0.0155 (11)	0.0054 (11)	−0.0404 (12)
C3	0.0570 (12)	0.0811 (15)	0.0517 (12)	−0.0177 (11)	0.0088 (10)	−0.0242 (11)
C4	0.0616 (12)	0.0603 (12)	0.0428 (10)	−0.0248 (10)	0.0020 (9)	−0.0059 (9)
C5	0.0632 (12)	0.0442 (10)	0.0373 (9)	−0.0175 (9)	0.0000 (8)	−0.0091 (8)
C6	0.0462 (9)	0.0418 (9)	0.0350 (9)	−0.0104 (7)	−0.0070 (7)	−0.0076 (7)
C7	0.0453 (9)	0.0353 (9)	0.0416 (9)	−0.0128 (7)	−0.0064 (7)	−0.0038 (7)
C8	0.0398 (9)	0.0380 (9)	0.0380 (9)	−0.0112 (7)	−0.0009 (7)	−0.0034 (7)
C9	0.0377 (8)	0.0442 (9)	0.0322 (8)	−0.0120 (7)	0.0031 (7)	−0.0093 (7)
C10	0.0355 (8)	0.0433 (9)	0.0364 (9)	−0.0119 (7)	−0.0018 (7)	−0.0109 (7)
C11	0.0442 (10)	0.0607 (12)	0.0448 (10)	−0.0055 (9)	0.0026 (8)	−0.0173 (9)
C12	0.0505 (11)	0.0570 (12)	0.0685 (14)	0.0073 (9)	−0.0104 (10)	−0.0224 (11)
C13	0.0622 (13)	0.0466 (11)	0.0648 (14)	−0.0111 (10)	−0.0239 (11)	−0.0051 (10)
C14	0.0574 (12)	0.0578 (12)	0.0477 (11)	−0.0199 (10)	−0.0058 (9)	0.0027 (9)
C15	0.0431 (10)	0.0503 (10)	0.0438 (10)	−0.0077 (8)	0.0043 (8)	−0.0079 (8)
C16	0.0371 (9)	0.0457 (9)	0.0434 (10)	−0.0104 (7)	−0.0020 (7)	−0.0124 (8)
C17	0.0701 (14)	0.0475 (11)	0.0566 (12)	−0.0120 (10)	−0.0104 (11)	−0.0076 (9)
C18	0.0883 (18)	0.0497 (12)	0.0921 (19)	−0.0035 (12)	−0.0234 (15)	−0.0188 (12)
N1	0.0489 (9)	0.0578 (10)	0.0633 (11)	−0.0226 (8)	−0.0079 (8)	−0.0049 (8)
N2	0.0519 (9)	0.0463 (9)	0.0558 (10)	−0.0185 (7)	−0.0095 (8)	−0.0084 (7)
N3	0.0475 (9)	0.0498 (9)	0.0628 (11)	−0.0097 (7)	−0.0080 (8)	−0.0208 (8)
O1	0.0418 (8)	0.1059 (14)	0.1195 (16)	−0.0223 (9)	0.0029 (9)	−0.0505 (12)
O2	0.0947 (13)	0.1034 (13)	0.0561 (10)	−0.0334 (11)	−0.0161 (9)	−0.0264 (9)
Se1	0.04099 (12)	0.07035 (15)	0.04790 (13)	−0.01791 (9)	0.00333 (8)	−0.00448 (9)

### Geometric parameters (Å, º)

**Table:**

C1—C2	1.370 (3)	C11—H11	0.9300
C1—C6	1.392 (3)	C12—C13	1.367 (3)
C1—H1	0.9300	C12—H12	0.9300
C2—C3	1.375 (3)	C13—C14	1.370 (3)
C2—H2	0.9300	C13—H13	0.9300
C3—C4	1.369 (3)	C14—C15	1.379 (3)
C3—H3	0.9300	C14—H14	0.9300
C4—C5	1.383 (3)	C15—H15	0.9300
C4—H4	0.9300	C16—N3	1.510 (2)
C5—C6	1.390 (3)	C16—C17	1.516 (3)
C5—H5	0.9300	C16—H16	0.9800
C6—C7	1.475 (3)	C17—C18	1.518 (3)
C7—C8	1.368 (3)	C17—H17A	0.9700
C7—N2	1.384 (2)	C17—H17B	0.9700
C8—C9	1.520 (2)	C18—H18A	0.9600
C8—Se1	1.839 (2)	C18—H18B	0.9600
C9—C10	1.523 (2)	C18—H18C	0.9600
C9—C16	1.534 (3)	N1—N2	1.267 (2)
C9—H9	0.9800	N1—Se1	1.8770 (19)
C10—C11	1.382 (3)	N3—O1	1.200 (2)
C10—C15	1.388 (3)	N3—O2	1.217 (2)
C11—C12	1.384 (3)		
			
C2—C1—C6	120.63 (19)	C13—C12—C11	120.4 (2)
C2—C1—H1	119.7	C13—C12—H12	119.8
C6—C1—H1	119.7	C11—C12—H12	119.8
C1—C2—C3	120.5 (2)	C12—C13—C14	119.5 (2)
C1—C2—H2	119.7	C12—C13—H13	120.2
C3—C2—H2	119.7	C14—C13—H13	120.2
C4—C3—C2	119.8 (2)	C13—C14—C15	120.6 (2)
C4—C3—H3	120.1	C13—C14—H14	119.7
C2—C3—H3	120.1	C15—C14—H14	119.7
C3—C4—C5	120.29 (19)	C14—C15—C10	120.59 (18)
C3—C4—H4	119.9	C14—C15—H15	119.7
C5—C4—H4	119.9	C10—C15—H15	119.7
C4—C5—C6	120.47 (18)	N3—C16—C17	108.74 (15)
C4—C5—H5	119.8	N3—C16—C9	108.55 (14)
C6—C5—H5	119.8	C17—C16—C9	112.31 (16)
C5—C6—C1	118.29 (18)	N3—C16—H16	109.1
C5—C6—C7	121.95 (16)	C17—C16—H16	109.1
C1—C6—C7	119.75 (16)	C9—C16—H16	109.1
C8—C7—N2	115.23 (17)	C16—C17—C18	114.36 (19)
C8—C7—C6	128.04 (16)	C16—C17—H17A	108.7
N2—C7—C6	116.72 (16)	C18—C17—H17A	108.7
C7—C8—C9	127.23 (16)	C16—C17—H17B	108.7
C7—C8—Se1	109.19 (12)	C18—C17—H17B	108.7
C9—C8—Se1	123.28 (13)	H17A—C17—H17B	107.6
C8—C9—C10	108.57 (14)	C17—C18—H18A	109.5
C8—C9—C16	110.27 (14)	C17—C18—H18B	109.5
C10—C9—C16	115.55 (14)	H18A—C18—H18B	109.5
C8—C9—H9	107.4	C17—C18—H18C	109.5
C10—C9—H9	107.4	H18A—C18—H18C	109.5
C16—C9—H9	107.4	H18B—C18—H18C	109.5
C11—C10—C15	118.18 (17)	N2—N1—Se1	110.66 (13)
C11—C10—C9	118.95 (16)	N1—N2—C7	117.79 (17)
C15—C10—C9	122.76 (16)	O1—N3—O2	124.35 (19)
C10—C11—C12	120.69 (19)	O1—N3—C16	117.82 (18)
C10—C11—H11	119.7	O2—N3—C16	117.82 (18)
C12—C11—H11	119.7	C8—Se1—N1	87.09 (8)
			
C6—C1—C2—C3	−0.8 (3)	C15—C10—C11—C12	−1.4 (3)
C1—C2—C3—C4	0.7 (4)	C9—C10—C11—C12	174.97 (17)
C2—C3—C4—C5	0.1 (3)	C10—C11—C12—C13	0.9 (3)
C3—C4—C5—C6	−0.9 (3)	C11—C12—C13—C14	0.2 (3)
C4—C5—C6—C1	0.8 (3)	C12—C13—C14—C15	−0.9 (3)
C4—C5—C6—C7	179.39 (17)	C13—C14—C15—C10	0.4 (3)
C2—C1—C6—C5	0.1 (3)	C11—C10—C15—C14	0.7 (3)
C2—C1—C6—C7	−178.58 (19)	C9—C10—C15—C14	−175.49 (17)
C5—C6—C7—C8	48.1 (3)	C8—C9—C16—N3	−172.07 (14)
C1—C6—C7—C8	−133.27 (19)	C10—C9—C16—N3	−48.5 (2)
C5—C6—C7—N2	−133.04 (18)	C8—C9—C16—C17	67.67 (19)
C1—C6—C7—N2	45.6 (2)	C10—C9—C16—C17	−168.76 (16)
N2—C7—C8—C9	−171.71 (16)	N3—C16—C17—C18	61.3 (2)
C6—C7—C8—C9	7.1 (3)	C9—C16—C17—C18	−178.51 (18)
N2—C7—C8—Se1	2.08 (19)	Se1—N1—N2—C7	0.6 (2)
C6—C7—C8—Se1	−179.08 (14)	C8—C7—N2—N1	−1.9 (2)
C7—C8—C9—C10	95.8 (2)	C6—C7—N2—N1	179.17 (16)
Se1—C8—C9—C10	−77.17 (17)	C17—C16—N3—O1	65.7 (2)
C7—C8—C9—C16	−136.66 (18)	C9—C16—N3—O1	−56.7 (2)
Se1—C8—C9—C16	50.36 (19)	C17—C16—N3—O2	−113.5 (2)
C8—C9—C10—C11	−97.56 (19)	C9—C16—N3—O2	124.09 (18)
C16—C9—C10—C11	137.99 (17)	C7—C8—Se1—N1	−1.40 (12)
C8—C9—C10—C15	78.6 (2)	C9—C8—Se1—N1	172.68 (15)
C16—C9—C10—C15	−45.8 (2)	N2—N1—Se1—C8	0.48 (14)

### Hydrogen-bond geometry (Å, º)

**Table:**

*D*—H···*A*	*D*—H	H···*A*	*D*···*A*	*D*—H···*A*
C16—H16···Se1	0.98	2.85	3.313 (3)	110
